# Ethical perceptions and learning engagement in AI-assisted translation: a behavioral psychology study of English learners

**DOI:** 10.3389/fpsyg.2026.1739478

**Published:** 2026-02-03

**Authors:** Lingli Zhang, Qian Li

**Affiliations:** School of Culture and Media, School of Marxism, Hubei Enshi College, Enshi, Hubei, China

**Keywords:** AI-assisted translation, English learners, ethical concerns, higher education, SPSS, student engagement, translation pedagogy

## Abstract

The rapid integration of artificial intelligence (AI) into language learning environments is reshaping translation pedagogy while raising important ethical and behavioral considerations. This research examines the structural relationship between students’ ethical perceptions of AI-assisted translation and their engagement in language learning activities. Data were gathered from 525 undergraduate English learners through a structured questionnaire addressing AI usage patterns, ethical perceptions (ethical awareness, academic integrity concern, algorithmic bias perception), and engagement behaviors. The dataset was analyzed using SPSS, applying descriptive statistics, ANOVA, correlation analysis, regression analysis, and cluster analysis. The results indicate that: (1) Ethical perceptions drive engagement: Ethical awareness and algorithmic bias perception are significant positive predictors of critical engagement, suggesting that recognizing system limitations stimulates deeper cognitive participation and “informed trust” rather than disengagement; (2) Integrity regulates reliance: Academic integrity concern is significantly and negatively associated with AI reliance, acting as a regulatory mechanism against over-dependence; and (3) Distinct learner profiles: Cluster analysis identified three distinct learner profiles: “Integrated Adopters” (High-Engagement/High-Trust), “Passive Dependents” (Low-Engagement/High-Reliance), and “Cautious Skeptics” (Low-Engagement/Low-Reliance). This research concludes that ethical literacy is a critical cognitive determinant of learning behavior. It offers empirical guidelines for shifting from prohibitive ethics to competency-based instruction that fosters responsible and engaged AI use in translation education.

## 1 Introduction

In the field of translation, artificial intelligence (AI) has emerged as a new toolset that enhances translation speed, accuracy, and accessibility (Ren, 2025). AI systems powered by large language models (LLMs) are increasingly capable of handling complex linguistic tasks, such as producing context-sensitive translations, supporting nuanced language comprehension, and assisting in advanced translation scenarios that demand creativity and adaptive decision-making (Gao et al., 2024). Consequently, English learners are increasingly drawing on AI tools to complete tasks such as initial drafting, text polishing, term selection, and quality checking in the context of in-class training, take-home assignments, and translation practice (Sahari et al., 2023). While AI improves efficiency and lowers the entry threshold for translation, it also reshapes students’ translation learning processes: rather than merely completing translation tasks in the traditional sense, students now process, correct, and further refine target texts through ongoing interaction with AI-generated outputs (Mohamed et al., 2024). This shift moves translation pedagogy beyond a sole emphasis on skill training toward an approach that additionally examines learners’ patterns of tool use, depth of cognitive engagement, and overall levels of learning engagement (Gaspari et al., 2015).

However, the pedagogical benefits of AI-assisted translation are also accompanied by notable ethical and behavioral challenges. On the one hand, academic integrity issues—such as plagiarism, overreliance (Campbell and Waddington, 2024), and substitutional task completion—may reinforce habitual patterns of AI use and in turn, alter students’ learning strategies and interest in classroom participation (Bittle and El-Gayar, 2025). On the other hand, algorithmic bias and unfair outputs—including cultural and register-related distortions, stereotypical representations, and unstable performance across specific text types—may shape students’ trust in the system and their intention to use it, thereby influencing their engagement and persistence in translation learning (Prates et al., 2020). In addition, whether students possess adequate ethical awareness and habits of critical evaluation will determine whether they passively accept AI-generated results or actively verify, compare, and reflect on them, leading to deeper forms of learning engagement (Goddard et al., 2012). From the perspective of behavioral psychology and engagement research, such ethical perceptions are not merely attitudinal judgments; they may influence learning behaviors through pathway mechanisms involving trust, reliance, and critical participation (Ali, 2023).

Although prior research has examined technology-enhanced language learning and learner engagement (Gaspari et al., 2015), direct and testable empirical evidence remains limited regarding how students’ ethical perceptions of AI-assisted translation are systematically associated with their usage behaviors and learning engagement in higher-education translation instruction, particularly in terms of quantitatively profiling learner differences. To address this gap, the present study surveyed undergraduate English learners (*N* = 525) and employed a questionnaire to measure ethical awareness, academic integrity concerns, perceptions of algorithmic bias, learning engagement and critical engagement, as well as AI trust and AI reliance. Using SPSS, we conducted descriptive statistics, correlation and Pearson correlation analyses, ANOVA, and cluster analysis to clarify the relational structure between ethical perceptions and engagement and to identify differentiated learner profiles. The findings are intended to provide empirical evidence and pedagogical implications for an integrated approach to translation instruction that combines skill development, ethical literacy, and responsible AI use.

### 1.1 Objectives

- To examine relationships between ethical perceptions of AI-assisted translation and student engagement (including critical engagement).
- To analyze how academic integrity concerns and perceived algorithmic bias are associated with AI reliance and AI trust, respectively, and how these relate to engagement.
- To identify learner profiles based on ethics-related perceptions and engagement-related behaviors and to derive pedagogical implications for ethical literacy in translation training.

Paper organization: This paper is organized as follows. Section 1 introduces the research background, identifies the research gap, and states the objectives. Section 2 reviews related work. Section 3 presents the hypothesis framework. Section 4 describes the methodology and data analysis procedures. Section 5 reports and discusses the results. Section 6 concludes with key findings, limitations, and implications.

## 2 Related works

The application of translation technology in translation education is not a new topic. From early computer-assisted translation (CAT) tools to neural machine translation (NMT), and more recently to generative systems represented by large language models (LLMs), translation classrooms have gradually shifted from an orientation toward “translation product output” to a focus on “process and decision-making.” Related research indicates that the most salient value of AI tools lies not merely in increasing speed, but in reshaping learners’ workflows and the structure of their cognitive tasks (Maier-Hein et al., 2022). Learners are required to make choices and judgments across multiple stages, including pre-editing, post-editing of machine output, maintaining stylistic consistency, and quality checking (Su and Yang, 2023). Accordingly, discussions of “technological competence” in translation education have expanded beyond training in software operations to encompass more complex process-oriented competences, such as post-editing evaluation, error diagnosis, understanding quality standards, and multi-round revision strategies (Eaton, 2022).

With the growing prevalence of generative AI in translation learning, ethical issues have become an important strand of research in translation education (Foltynek et al., 2023). First, academic integrity concerns have become more pronounced in a context where the substitutability of tools has markedly increased (Sallam et al., 2024). Such concerns include undisclosed external generation, blurred authorship of assignments, and the risk that learners replace learning processes with mere output production. Prior studies commonly emphasize that integrity risks are associated not only with whether tools are used, but also with how they are used (Blahopoulou and Ortiz-Bonnin, 2025). For instance, when learners employ AI as a means of substitutional task completion, this often coincides with reduced reflection, fewer instances of error diagnosis, and weaker self-regulation (Balalle and Pannilage, 2025). Consequently, many pedagogical discussions advocate establishing transparent disclosure and citation norms, clarifying acceptable boundaries of assistance, and embedding ethical guidelines within course assessment systems (Sebastião and Dias, 2025).

Second, algorithmic bias and unfair outputs have long been central concerns in language technology research and are particularly salient in translation contexts. Research suggests that training data and model architectures may produce systematic deviations in cultural pragmatics, gender and group representation, language variation, and register appropriateness (Jain, 2024). In addition, performance fluctuations across text types, domains, and low-resource language settings may amplify risks of instability and unfairness (Farkas and Németh, 2022). In translation teaching, such biases not only affect translation quality but may also shape learners’ calibration of trust in the system and their willingness to use it (Karastergiou and Diamantopoulos, 2024), thereby indirectly influencing the learning process (Dilmaghani et al., 2019). Beyond integrity and bias, responsible use also encompasses issues such as data privacy, copyright and compliance regarding training data, and accountability for “hallucinations” or misinformation (King, 2025). Overall, ethical discussions are increasingly moving from the mere articulation of norms toward competence development—treating ethical awareness, risk identification, and verification strategies as integral components of translation competence rather than external constraints.

In behavioral psychology and educational technology research, learners’ trust in and reliance on systems are often regarded as key mechanism variables shaping the effectiveness of technology use (Nowotny, 2024). Studies show that learners’ subjective judgments about a tool’s reliability influence whether they verify outputs and whether they retain autonomous decision-making (Pedro, 2012). In AI-assisted translation contexts, excessive trust may lead learners to adopt outputs directly and reduce revision and reflection; conversely, insufficient trust may result in avoidance or excessive time spent on low-yield checking, thereby diminishing learning efficiency and participation experiences (Ibarra-Sáiz et al., 2025). Hence, the key issue is not whether “high trust” or “low trust” is preferable, but whether trust appropriately matches system capability—namely, trust calibration (Lee and See, 2004).

Corresponding to trust, reliance reflects the extent to which learners treat the tool as a primary pathway for task completion (Zhang et al., 2020). Research generally suggests that reliance is not inherently negative: under appropriate task division, tools can handle mechanical or repetitive operations, allowing learners to allocate resources to higher-order pragmatic, stylistic, and discourse-level decision-making (Başer and Aral, 2024). However, when reliance manifests as substitutional task completion, it may undermine learners’ monitoring and reflective behaviors (Do Carmo and Moorkens, 2020). Therefore, ethical perceptions may affect the quality of learning engagement by shaping trust and reliance, particularly influencing “critical engagement,” defined as the degree to which learners verify, diagnose, compare, and reflect on AI outputs (Parasuraman and Manzey, 2010). In instructional contexts, critical engagement is commonly understood to be associated with more responsible use of AI: it helps learners identify and mitigate risks arising from bias or inappropriate use, and it reflects learners’ proactive monitoring and self-regulation when working with tool-based assistance (Wu, 2024).

Although prior research has addressed translation technology teaching, ethical governance, and learning behaviors, the existing evidence remains insufficient in several respects. First, a substantial portion of AI-related research in translation education remains focused on tool effects, attitudes, or adoption intentions, with limited systematic quantitative testing of the structural relationships among “ethical perceptions—trust—learning engagement (including critical engagement).” Second, learner differences are often presented through descriptive categorization or case-based analyses, with limited multivariate statistical profiling to explain why learners who use AI in similar ways may nonetheless exhibit markedly different participation patterns and learning outcomes. Third, within higher-education contexts, English learners constitute an important group of translation learners, yet more representative samples and more comprehensive analytical frameworks are still needed to clarify how their AI-use behaviors and ethical perceptions jointly shape learning engagement.

In light of these gaps, the present study integrates ethical perceptions, trust and reliance mechanisms, and learning engagement in a translation teaching context, and further identifies distinct participation patterns through learner profiling. The study aims to provide actionable empirical evidence and pedagogical insights for an integrated approach to translation education that combines skill development, ethical literacy, and responsible AI use.

## 3 Hypothesis framework

The hypothesis framework investigates ethical attitudes, academic integrity, algorithmic bias, ethical awareness, learner profiles, and ethical literacy, focusing on their overall impact on student engagement, trust, and responsible usage of AI-assisted translation tools.

*H1*: Ethical awareness and algorithmic bias perception are positively associated with student engagement in translation learning.

Students with stronger ethical awareness tend to show more engaged learning behaviors. Learners who report stronger perceptions/awareness of algorithmic bias may also engage more actively with AI-assisted translation tasks, potentially through closer monitoring and involvement.

*H2*: Concerns about academic integrity are negatively associated with students’ reliance on AI-assisted translation tools.

Students who are more concerned about academic integrity may intentionally reduce dependence on AI tools to avoid misconduct risks and maintain originality in learning tasks.

*H3*: Algorithmic bias perception is positively associated with students’ trust in AI-assisted translation systems.

Students’ bias-related perceptions are linked to how they evaluate AI systems. Higher bias perception scores are expected to be associated with higher reported trust in AI-assisted translation tools.

*H4*: Students with higher ethical awareness demonstrate greater critical engagement in translation learning compared to those with lower ethical awareness.

Ethically aware students are more likely to verify, evaluate, and revise AI-generated outputs, reflecting deeper critical engagement.

*H5*: Distinct learner profiles (high-engagers, moderate-engagers, low-users) show significant differences in their ethical concerns regarding AI-assisted translation.

Because learner profiles reflect different patterns of engagement, reliance, and trust, they are expected to differ in academic integrity concern and algorithmic bias perception across groups.

*H6*: A composite ethical-perception factor is positively associated with students’ critical engagement in AI-assisted translation learning.

An overall ethical-perception tendency (derived from ethical awareness, integrity concern, and algorithmic bias perception) is expected to correspond to more frequent critical evaluation of AI outputs.

## 4 Research methodology

**Figure 1:**
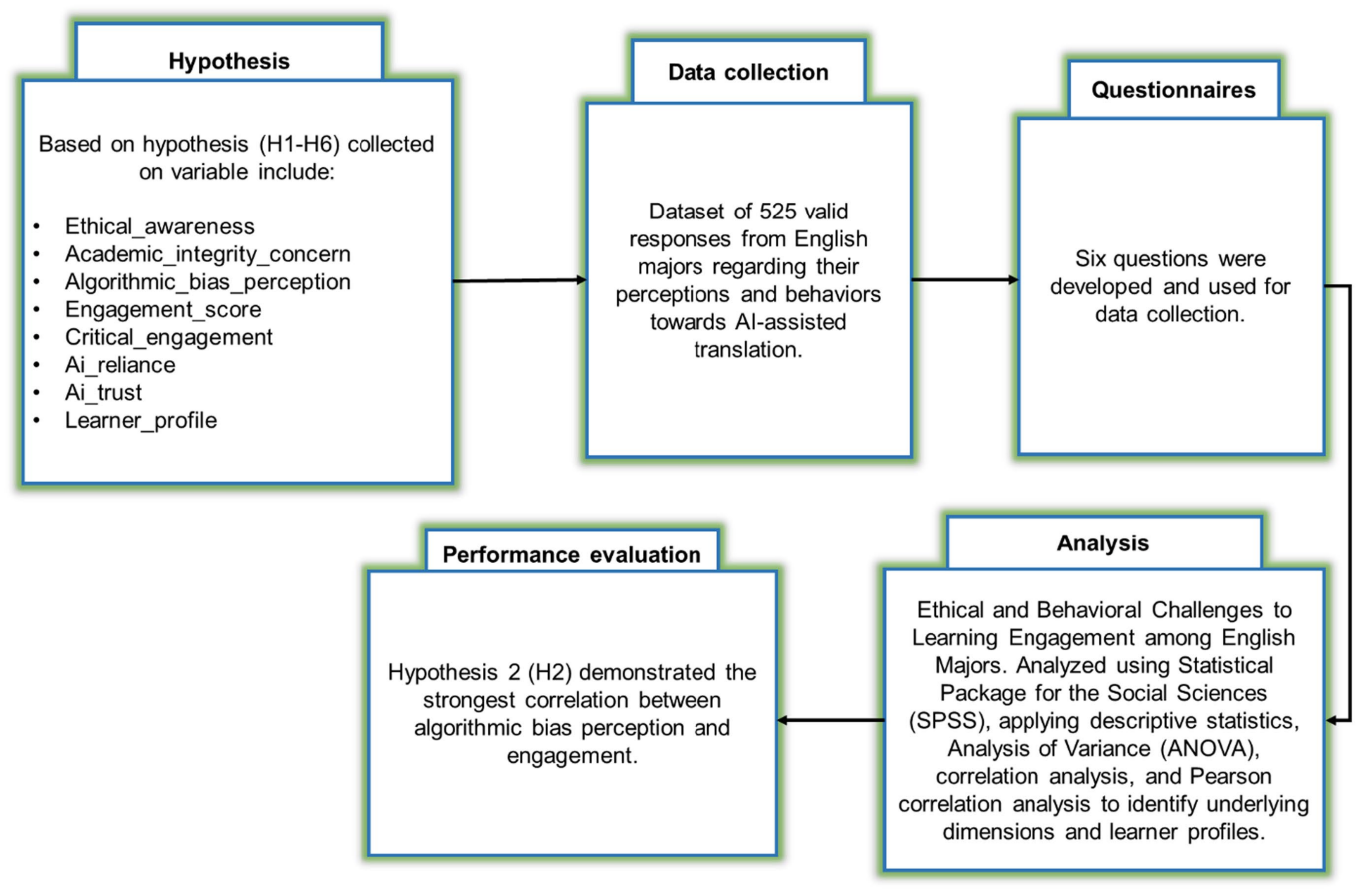
The methodology flow diagram.

To investigate ethical concerns, learner profiles, and engagement patterns in AI-assisted translation learning, researchers used a structured questionnaire and SPSS, applying descriptive statistics (DS), ANOVA, correlation analysis, and Pearson correlation (PC) analysis to identify underlying dimensions and learner profiles. Figure 1 displays the methodology flow diagram of learning engagement among English learners.

### 4.1 Data collection

This research used a dataset of 525 valid responses from English learners regarding their perceptions and behaviors towards AI-assisted translation. The research measured eight normalized numeric variables: ethical awareness, academic integrity concern, algorithmic bias perception, engagement score, critical engagement, AI dependence, AI trust, and learner profile. The factors assessed students’ ethical awareness, integrity concerns, perceptions of algorithmic bias, engagement levels, reliance on AI tools, faith in AI, and learner clustering. Table 1 provides the sample of dataset collection. As shown in Table 1.

**Table 1:** Variable definitions and coding.

Construct (abbr.)	Role in hypotheses	Operational definition	No. of items	Response format	Scoring and transformation	Reliability
Ethical awareness (EA)	IV	Awareness of ethical issues in AI-assisted translation learning	1	5-point Likert agreement (1 = strongly disagree, 5 = strongly agree)	Item score (1–5); higher = higher ethical awareness	*α* = 0.86
Academic integrity concern (AIC)	IV	Concern about plagiarism and academic misconduct when using AI tools	1	5-point Likert agreement (1 = strongly disagree, 5 = strongly agree)	Item score (1–5); higher = stronger academic integrity concern	*α* = 0.79
Algorithmic bias perception (ABP)	IV	Perceived bias in AI translation outputs	1	5-point Likert agreement (1 = strongly disagree, 5 = strongly agree)	Item score (1–5); higher = stronger perceived algorithmic bias	*α* = 0.82
Engagement (ENG)	DV	Behavioral engagement in translation learning activities	1	5-point frequency (1 = never, 5 = always)	Item score (1–5); higher = higher learning engagement	*α* = 0.88
Critical engagement (CENG)	DV	Verification, reflection, and evaluation of AI outputs	1	5-point frequency (1 = never, 5 = always)	Item score (1–5); higher = deeper critical engagement	*α* = 0.84
AI reliance (AIR)	Mediator and outcome	Extent of dependence on AI tools for translation tasks	1	5-point frequency (1 = never, 5 = always)	Item score (1–5); higher = stronger AI reliance	*α* = 0.89
AI trust (AIT)	Mediator and outcome	Perceived reliability of AI-assisted translation systems	1	5-point Likert agreement (1 = strongly disagree, 5 = strongly agree)	Item score (1–5); higher = greater AI trust	*α* = 0.85
Learner profile	Grouping variable	Cluster membership derived from ENG, AIR, AIT	3 inputs	Categorical (cluster membership)	k-means clustering (*k* = 3) using ENG, AIR, and AIT; report cluster labels and centers	

### 4.2 Measures

This study employed a quantitative design using a structured questionnaire to assess English learners’ perceptions and behaviors related to AI-assisted translation learning. For hypothesis testing, survey responses were summarized at the construct level to form seven continuous indices: ethical awareness, academic integrity concern, algorithmic bias perception, engagement score, critical engagement, AI reliance, and AI trust. All continuous indices were scaled to the range of 0 to 1 to facilitate comparability across constructs, where higher values indicate higher levels of the corresponding construct (see Table 1 for variable definitions and coding).

#### 4.2.1 Ethical perceptions of AI

Ethical perceptions were operationalized using three indices: ethical awareness (awareness of ethical issues in AI-assisted translation), academic integrity concern (concerns regarding plagiarism or dishonest use of AI tools), and algorithmic bias perception (perceived fairness and potential bias in AI outputs). Higher values reflect stronger ethical awareness, greater integrity concerns, and stronger perceived bias, respectively.

#### 4.2.2 Learning engagement

Learning engagement was represented by two indices. Engagement score captured general behavioral involvement in translation-related learning activities, whereas critical engagement captured deeper cognitive engagement such as verifying and reflecting on AI-generated outputs. Higher values indicate higher general engagement and stronger critical engagement.

#### 4.2.3 AI usage behaviors

AI usage behaviors were captured by two indices: AI reliance (the extent to which students depend on AI tools) and AI trust (students’ perceived reliability and accuracy of AI systems). Higher values indicate stronger reliance and greater trust, respectively.

#### 4.2.4 Learner profile

To characterize heterogeneity in learner patterns, learner profile was derived using k-means clustering (*k* = 3) based on engagement score, AI reliance, and AI trust. The resulting cluster membership was used as a categorical grouping variable in subsequent analyses.

### 4.3 Analysis methods

The analysis uses correlation, such as PC, DS, and ANOVA, to investigate the links between ethical concerns, trust, engagement, and learner profiles, demonstrating that ethics has a major influence on AI-assisted translation behaviors, which are analyzed using SPSS.

#### 4.3.1 Correlation

The correlation analysis in this study examines how students’ ethical perceptions of AI-assisted translation are associated with engagement- and usage-related outcomes. Specifically, academic integrity concern is expected to be negatively associated with AI reliance, as students who are more concerned about originality and appropriate conduct may avoid depending heavily on AI tools. In addition, algorithmic bias perception is examined in relation to students’ trust and engagement to determine whether bias-related perceptions are significantly associated with confidence in AI-supported learning activities. Finally, ethical awareness is expected to be positively associated with critical engagement, reflecting more frequent verification, evaluation, and reflective revision when interacting with AI-generated translations.

#### 4.3.2 Pearson correlation (PC)

The Pearson correlation assesses the strength and direction of a linear relationship between two continuous variables. In this research context, this statistically advances the hypothesis that ethical concerns are related to learning behaviors. A positive PC value (e.g., *r* > 0) indicates that higher ethical awareness correlates to higher engagement. A negative PC value (e.g., *r* < 0) would confirm that considerations of integrity or algorithmic bias lessen consideration for reliance or trust. A statistically significant result indicating a valid relationship (i.e., *p* < 0.05) is expected.

#### 4.3.3 Descriptive statistics in regression

Prior to conducting regression, DS assists in summarizing the independent variables of interest: ethical concerns, ethical awareness, AI reliance, trust, and engagement. In SPSS, DS provides the Ms., standard deviations, minimums, and maximums for each dependent variable. The research investigates the impact of ethical judgments of AI-assisted translation on student engagement, dependence, trust, and learning practices. It uses DS to provide context for interpreting coefficients and significance tests. The central tendency measures students’ average participation or ethical awareness, while the dispersion measures perceptional variability.

#### 4.3.4 Analysis of variance (ANOVA)

ANOVA will be applied in this research to help investigate whether learner profiles differ significantly with respect to ethical concerns, engagement, and trust. ANOVA, which is motivated by the variance between groups relative to within-group variance, is a tool for determining whether at least one of the groups’ Ms. are significantly different (*p* < 0.05).

## 5 Results

This section includes descriptive, correlation, regression, and ANOVA results that explain how ethical attitudes, academic integrity, and bias affect engagement, AI reliance, and learner profiles in translation pedagogy.

### 5.1 Preliminary analyses

**Figure 2:**
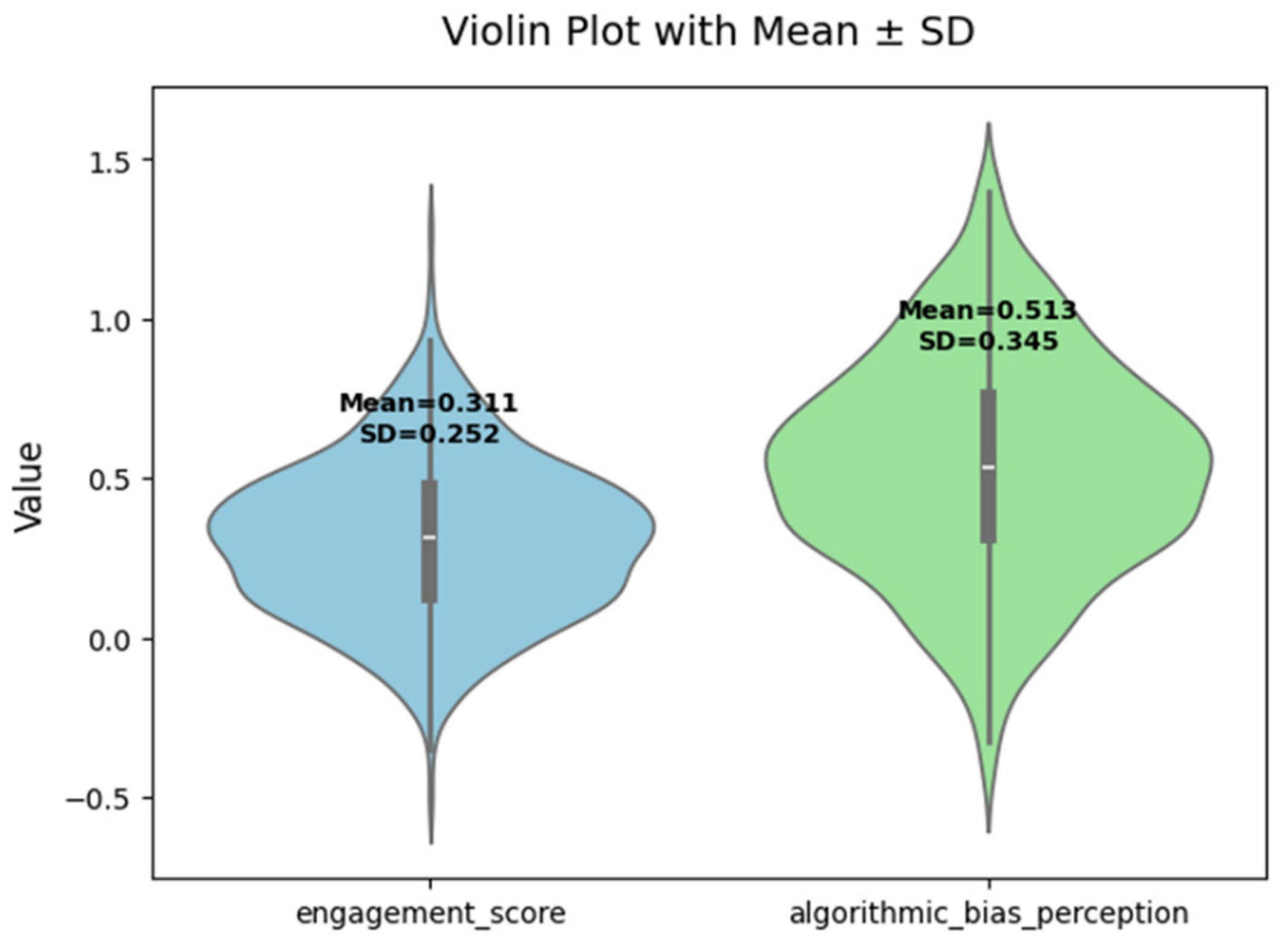
Comparative distribution of engagement score and algorithmic bias perception.

A total of 525 valid responses were included in the analysis. Overall, students demonstrated a moderate level of engagement in translation-related learning tasks (engagement score: *M* = 0.311, SD = 0.252). Students’ perceptions of algorithmic bias were also at a moderate-to-high level (algorithmic bias perception: *M* = 0.513, SD = 0.345), indicating that participants were relatively sensitive to potential unfairness or bias in AI translation outputs. Regarding AI-related attitudinal variables, the overall level of trust in AI-assisted translation systems was relatively low (AI trust: *M* = 0.253, SD = 0.243). This pattern suggests that, despite recognizing potential bias-related issues, students generally reported limited confidence in AI-supported translation activities. In addition, critical engagement was at a moderate level (critical engagement: *M* = 0.309, SD = 0.261), suggesting that students were not merely passive users of AI tools but exhibited some degree of reflective and evaluative engagement when interacting with AI-generated translations. As shown in Table 2 and Figure 2.

**Table 2:** Descriptive statistics of key variables.

DS	*M*	S.D	*N*
AIT	0.253	0.243	525
ABP	0.513	0.345	525
ENG_score	0.311	0.252	525
CE	0.309	0.261	525
REGR-FS	0.000	1.000	525

### 5.2 Correlations among key variables

Correlation analyses indicated that ethical awareness was positively associated with overall engagement (*r* = 0.430, *p* < 0.001), suggesting that students with stronger ethical awareness tended to participate more actively in translation learning. Ethical awareness was also strongly and positively correlated with algorithmic bias perception (*r* = 0.622, *p* < 0.001), implying that ethically aware students were more likely to recognize or attend to bias-related issues in AI translation outputs. Furthermore, algorithmic bias perception was positively correlated with engagement (*r* = 0.355, *p* < 0.001). This result suggests that students who reported stronger bias-related perceptions also tended to exhibit higher engagement in learning activities, potentially reflecting more frequent monitoring, checking, or comparative evaluation during AI-assisted translation tasks. As shown in Table 3.

**Table 3:** Pearson correlation matrix among key variables.

Variable	1	2	3	4	5	6	7
1. EA	—						
2. IC	0.027	—					
3. BP	−0.115**	−0.083	—				
4. AIT	*	*	0.605**	—			
5. AIR	*	−0.665**	*	*	—		
6. ENG	0.437**	−0.001	0.355**	*	*	—	
7. CENG	*	*	*	*	*	0.356**	—

*Indicates *p* < 0.05, and **Indicates *p* < 0.01.

### 5.3 Regression models

#### 5.3.1 Predicting AI trust from algorithmic bias perception

**Figure 3:**
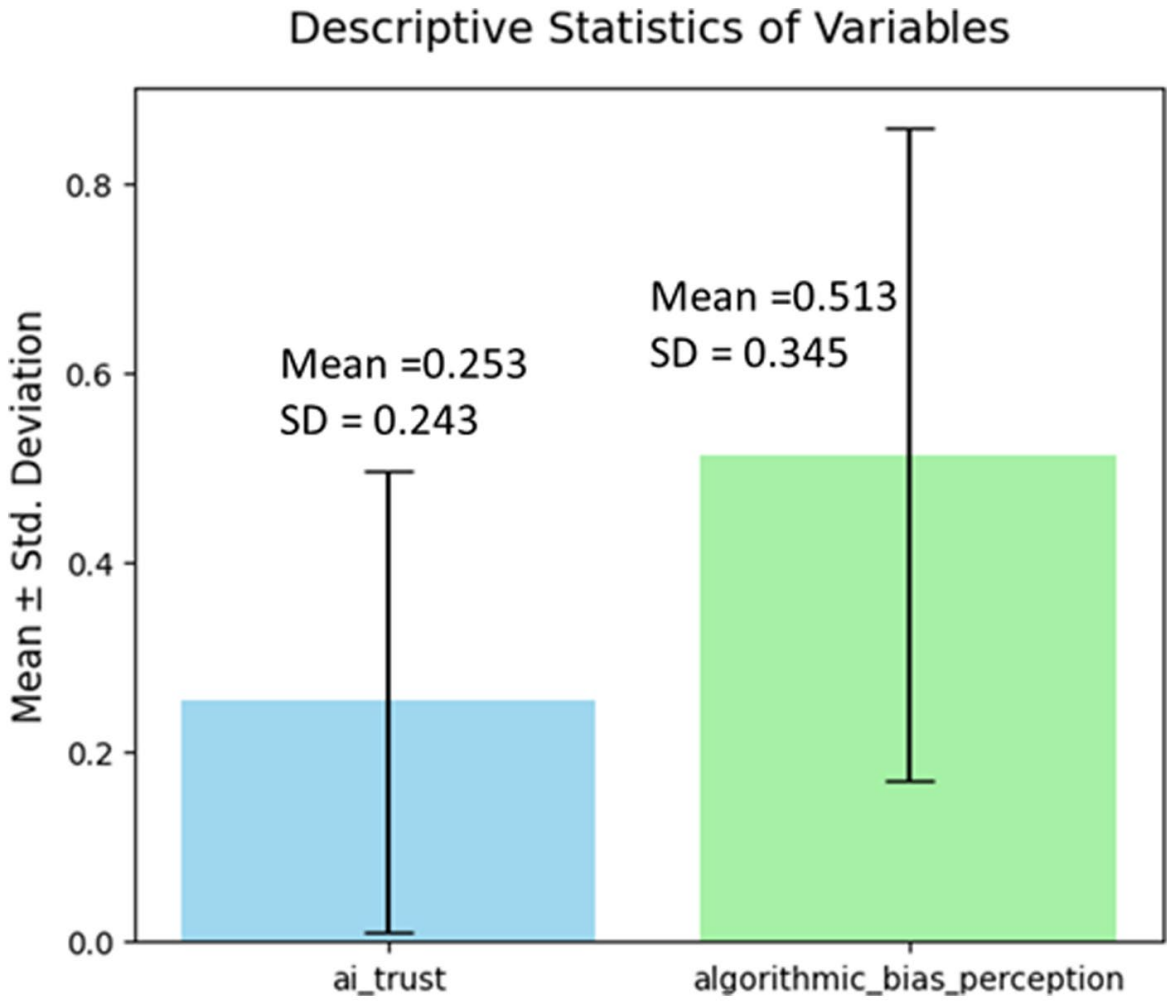
Discrepancy between the levels of algorithmic bias perception and AI trust.

A linear regression model indicated that algorithmic bias perception significantly predicted AI trust. The model demonstrated a strong fit (*R* = 0.605, *R*^2^ = 0.366, adjusted *R*^2^ = 0.365, *F*(1, 523) = 301.561, *p* < 0.001), showing that algorithmic bias perception explained 36.6% of the variance in AI trust. At the coefficient level, algorithmic bias perception was a significant positive predictor of AI trust (*B* = 0.427, *β* = 0.605, *t* = 17.366, *p* < 0.001), indicating that higher bias perception scores were associated with higher levels of reported trust. As shown in Figure 3.

#### 5.3.2 Predicting engagement from algorithmic bias perception

Algorithmic bias perception also significantly predicted overall engagement. The regression model was statistically significant (*R* = 0.355, *R*^2^ = 0.126, adjusted *R*^2^ = 0.124, *F*(1, 523) = 75.486, *p* < 0.001), explaining 12.6% of the variance in engagement. This finding suggests that bias-related perceptions are not only attitudinal in nature but are also systematically associated with students’ learning engagement.

#### 5.3.3 Predicting critical engagement from algorithmic bias perception

A regression model further showed that algorithmic bias perception significantly predicted critical engagement [*R*^2^ = 0.127, *F*(1, 523) = 75.953, *p* < 0.001]. Algorithmic bias perception was a significant positive predictor of critical engagement (*B* = 0.093, β = 0.356, *t* = 8.715, *p* < 0.001). This result indicates that students who reported stronger bias-related perceptions were more likely to engage critically and reflectively when using AI-assisted translation tools. As shown in Table 4.

**Table 4:** Summary of regression analysis for predicting trust and engagement.

	Model 1	Model 2	Model 3
Outcome Variable	AI Trust	Engagement	Critical engagement
Predictor	*Bias perception*	*Bias perception*	*Ethical factor score*

	*β*	*t*	*β*	*t*	*β*	*t*
Standardized coeff.	0.605	17.37***	0.355	8.69***	0.356	8.72***
*R* ^2^	0.366		0.126		0.127	
Adjusted *R*^2^	0.365		0.124		0.125	
FValue	301.56***		75.49***		75.95***	

### 5.4 Group differences

To examine whether critical engagement differed across levels of ethical awareness, a one-way ANOVA was conducted. The results indicated a significant effect of ethical awareness level on critical engagement [*F*(2, 522) = 60.255, *p* < 0.001]. *Post hoc* comparisons using Tukey’s HSD test further showed that students in the high ethical awareness group demonstrated significantly higher critical engagement than those in the medium and low ethical awareness groups. Specifically, the mean level of critical engagement was approximately 0.514 in the high group, 0.286 in the medium group, and 0.121 in the low group, with both high–low and high–medium differences reaching statistical significance. Overall, these group differences are consistent with the correlational and regression findings, suggesting that stronger ethical awareness is associated with more frequent verification, evaluation, and reflective revision behaviors during AI-assisted translation learning.

### 5.5 Construct exploration

#### 5.5.1 Overview of cluster profiles

Cluster analysis identified three distinct learner profiles (cluster sizes = 181, 143, and 201, respectively). The three profiles were clearly differentiated in their cluster centers across key indices, including engagement, academic integrity concern, algorithmic bias perception, AI reliance, and AI trust. For example, Profile 2 showed the highest levels of engagement and AI trust (engagement score ≈ 0.433; AI trust ≈ 0.444), whereas Profile 1 exhibited higher academic integrity concern and algorithmic bias perception (academic integrity concern ≈ 0.629; algorithmic bias perception ≈ 0.763) and the lowest AI reliance (AI reliance ≈ 0.125).

#### 5.5.2 Profile differences (ANOVA and *post hoc* comparisons)

One-way ANOVAs indicated significant differences across learner profiles for multiple variables, including academic integrity concern [*F*(2, 522) = 13.839, *p* < 0.001], algorithmic bias perception [*F*(2, 522) = 39.776, *p* < 0.001], AI reliance [*F*(2, 522) = 48.521, *p* < 0.001], and AI trust [*F*(2, 522) = 53.338, *p* < 0.001]. Tukey *post hoc* tests further suggested a consistent pattern of differentiation across profiles. For instance, with respect to algorithmic bias perception, Profile 1 scored significantly higher than Profile 3, and Profile 2 also scored significantly higher than Profile 3.

**Figure 4:**
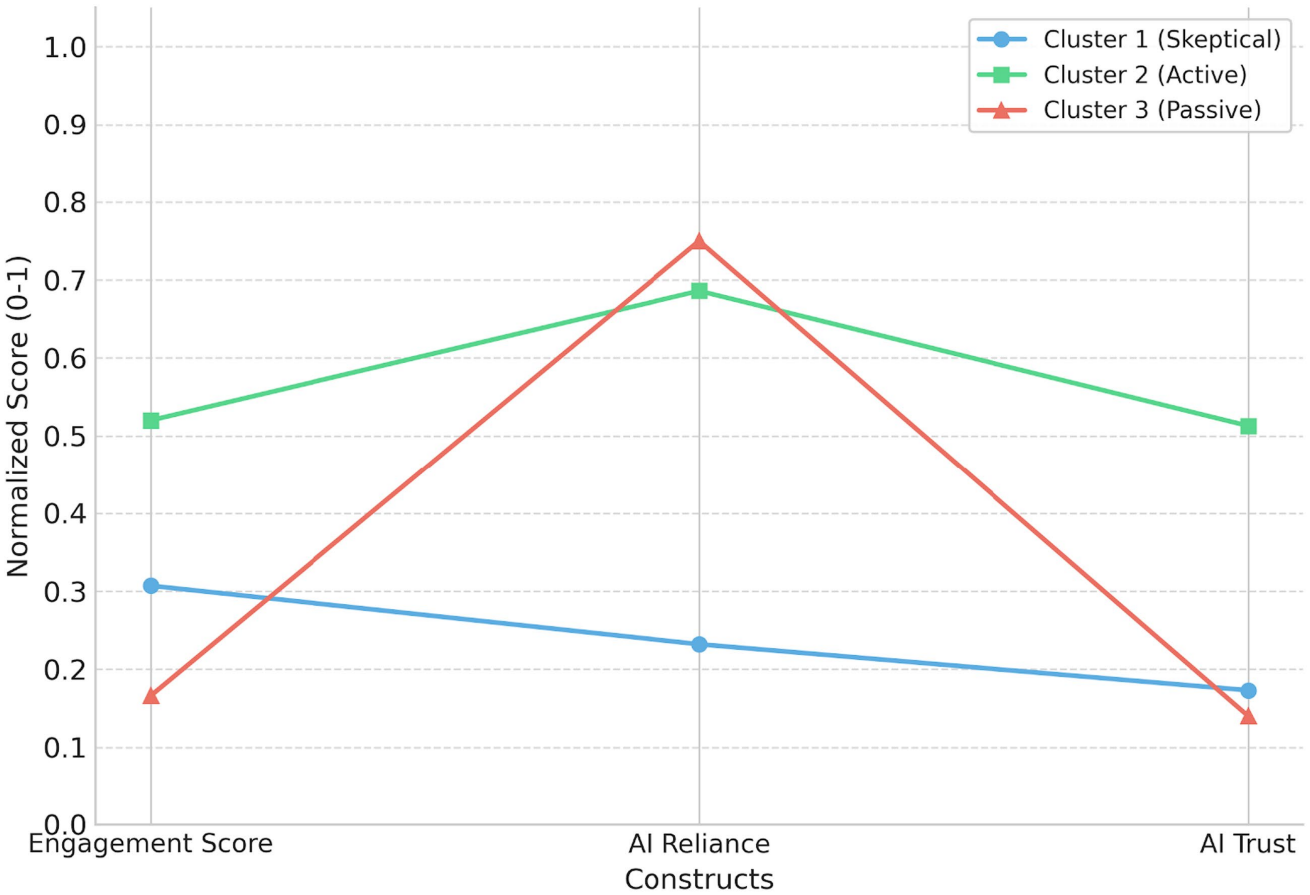
Learner profiles based on final cluster centers for engagement, AI reliance, and AI trust.

Taken together, the three profiles can be interpreted as: (a) a high bias-sensitivity/high integrity concern/low reliance profile, (b) a high engagement/high trust profile, and (c) a comparatively lower sensitivity/lower trust or lower engagement profile. These profile-based results provide a structured basis for discussing heterogeneous patterns in how students use and evaluate AI-assisted translation tools. As shown in Table 5 and Figure 4.

**Table 5:** Learner profiles identified via K-means clustering.

Cluster profile	*N*	Engagement (Center)	AI reliance (Center)	AI trust (Center)	Interpretation
Cluster 1	181	0.307	0.232	0.173	Skeptical/Low-reliance users
Cluster 2	143	0.52	0.686	0.513	High-engagement/Trusting users
Cluster 3	201	0.166	0.75	0.14	Disengaged/Dependent users

### 5.6 Composite ethical perception and critical engagement

To test Hypothesis 6, which posits that an overarching ethical mindset is associated with deeper cognitive involvement, a Principal Component Analysis (PCA) was first conducted to derive a composite factor from the three ethical indicators: ethical awareness, academic integrity concern, and algorithmic bias perception. The analysis extracted a single dominant component (eigenvalue = 1.155), which explained 38.51% of the total variance. The factor loadings revealed that ethical awareness (0.622) and academic integrity concern (0.494) were the primary contributors to this composite construct, reflecting a consolidated “ethical consciousness” among students.

Subsequently, a linear regression analysis was performed using this composite factor score as the independent variable and critical engagement as the dependent variable. The regression model was statistically significant, *F*(1, 523) = 75.95, *p* < 0.001. The results indicated that the composite ethical factor accounted for 12.7% of the variance in critical engagement (R2 = 0.127). Crucially, the composite factor was a significant positive predictor of critical engagement (*β* = 0.356, t = 8.72, p < 0.001). These findings support H6, suggesting that students with a holistic ethical mindset—characterized by high awareness and integrity concerns—are significantly more likely to engage in critical verification and reflection when using AI translation tools. As shown in Table 6.

**Table 6:** Factor loadings for the composite ethical perception construct.

Item/Indicator	Factor loading
Ethical awareness	0.622
Academic integrity concern	0.494
Algorithmic bias perception	−0.115
Eigenvalue	1.155
Variance explained	38.51%

## 6 Discussion

This study investigated the structural relationships between ethical perceptions, mechanism variables (trust and reliance), and learning engagement in the context of AI-assisted translation. The findings provide empirical evidence that ethical literacy functions as a critical cognitive determinant that shapes students’ interaction patterns with AI tools.

### 6.1 Ethical awareness as a cognitive driver of critical engagement

The results support Hypothesis 1 (H1) and Hypothesis 6 (H6), demonstrating that ethical awareness is a significant positive predictor of student engagement, specifically critical engagement. This finding challenges the view that ethical constraints merely inhibit technology use. Instead, the data suggest that ethical awareness functions as a cognitive prerequisite for active evaluation. Students with higher ethical awareness are more likely to engage in “human-in-the-loop” behaviors—such as verifying terminology and correcting errors—rather than passively accepting AI outputs. This aligns with the ANOVA results for Hypothesis 4 (H4), where high-awareness students exhibited significantly higher levels of critical evaluation compared to their lower-awareness peers. Consequently, ethical literacy appears to facilitate a shift from passive consumption to active quality control in the translation process.

### 6.2 The regulatory function of academic integrity concern

The significant negative correlation between academic integrity concern and AI reliance (Hypothesis 2), indicates that integrity concerns operate as a regulatory mechanism on behavioral dependence. Students who prioritize authorship and originality tend to restrict their frequency and intensity of AI usage to avoid potential misconduct. While this regulation preserves academic honesty, the strong inverse relationship suggests a potential trade-off: excessive apprehension regarding integrity may inhibit the instrumental use of AI for legitimate auxiliary tasks, such as lexical retrieval or syntactic polishing. This points to the necessity of pedagogical frameworks that clearly distinguish between “improper substitution” and “legitimate assistance,” enabling students to utilize the technology without violating integrity standards.

### 6.3 The phenomenon of informed trust

A notable finding regarding Hypothesis 3 (H3) is the positive association between algorithmic bias perception and AI trust. This result diverges from the intuitive expectation that perceiving flaws would diminish trust. This observed relationship can be interpreted as “informed trust” or “conditional trust.” Students who frequently utilize AI tools likely develop a more nuanced understanding of both the system’s utility and its limitations (including bias). Therefore, their reported “trust” may reflect confidence in the tool’s functional utility and their own ability to manage its output, rather than a belief in the system’s infallibility. The regression results indicate that bias perception significantly predicts engagement, suggesting that the recognition of algorithmic limitations stimulates active cognitive processing rather than disengagement.

### 6.4 Heterogeneity in learner behavioral profiles

The cluster analysis (Hypothesis 5) revealed three distinct behavioral patterns that underscore the heterogeneity of the student population.

Cluster 1 (Skeptical/Low-Reliance Users) exhibits a profile of “cautious avoidance,” marked by moderate engagement but low reliance and trust. This group appears to prioritize traditional translation methods, possibly due to unresolved ethical concerns or skepticism regarding AI capabilities.

Cluster 2 (High-Engagement/Trusting Users) represents a profile of “integrated adoption,” where students combine high levels of trust and reliance with high cognitive engagement. This group effectively leverages the tool’s affordances while maintaining active participation.

Cluster 3 (Disengaged/Dependent Users) presents a profile of “passive dependence,” characterized by high reliance (0.750) but minimal engagement (0.166) and low trust (0.140). This pattern suggests a utilitarian approach where students use the tool for task completion despite lacking confidence in its quality, representing a high-risk group for learning loss.

## 7 Conclusion

This study examined the impact of ethical perceptions on the learning engagement of English learners in AI-assisted translation. The quantitative analysis of 525 undergraduates confirms that ethical perceptions are active drivers of learning behavior. Specifically, ethical awareness and algorithmic bias perception significantly predict critical engagement, suggesting that recognizing system limitations stimulates deeper cognitive processing rather than disengagement. Conversely, academic integrity concerns function as a regulatory mechanism that curbs excessive AI reliance. Furthermore, the identification of three distinct learner profiles—Integrated, Passive-Dependent, and Cautious—reveals that students utilize AI through complex configurations of trust and reliance, underscoring the heterogeneity of the learner population.

Theoretically, this research extends behavioral models in translation education by situating ethical constructs as central determinants of engagement rather than peripheral constraints. Pedagogically, the findings advocate for a shift from prohibitive ethics to competency-based instruction. Educators should leverage bias perception as a catalyst for critical AI literacy, help dependent learners calibrate their reliance to avoid cognitive passivity, and establish clear norms that distinguish legitimate assistance from plagiarism. Ultimately, the goal is to foster “informed trust,” where students use AI efficiently while maintaining critical oversight.

The generalizability of this study is limited by its single-institution sample and reliance on self-reported measures, which may be subject to social desirability bias. Future research should corroborate these findings using objective translation performance metrics and experimental designs. Additionally, longitudinal studies are recommended to track how ethical perceptions and behavioral profiles evolve as students develop greater technological proficiency.

## Data Availability

The raw data supporting the conclusions of this article will be made available by the authors, without undue reservation.

## References

[ref1] Ali J. K. M. (2023). Benefits and challenges of using ChatGPT: an exploratory study on English language program. Univ. Bisha J. Human. 2, 629–641.

[ref2] Balalle H. Pannilage S. (2025). Reassessing academic integrity in the age of AI: a systematic literature review on AI and academic integrity. Soc. Sci. Human. Open 11:101299.

[ref3] Başer Z. Aral M. (2024). Perspectives of translation students on artificial intelligence-based translation tools. Kırıkkale Üniversitesi Sosyal Bilimler Dergisi 14, 39–55.

[ref4] Bittle K. El-Gayar O. (2025). Generative AI and academic integrity in higher education: a systematic review and research agenda. Information 16:296.

[ref5] Blahopoulou J. Ortiz-Bonnin S. (2025). Student perceptions of ChatGPT: benefits, costs, and attitudinal differences between users and non-users toward AI integration in higher education. Educ. Inf. Technol., 1–24.

[ref6] Campbell C. Waddington L. (2024). Academic integrity strategies: student insights. J. Acad. Ethics 22, 33–50.

[ref7] Dilmaghani S. Brust M. R. Danoy G. Cassagnes N. Pecero J. Bouvry P. (2019). “Privacy and security of big data in AI systems: a research and standards perspective” in In 2019 IEEE international conference on big data (big data) (IEEE), 5737–5743.

[ref8] Do Carmo F. Moorkens J. (2020). “Differentiating editing, post-editing and revision” in Translation revision and post-editing (Routledge), 35–49.

[ref9] Eaton S. E. (2022). The academic integrity technological arms race and its impact on learning, teaching, and assessment. Can. J. Learn. Technol. 48, 1–9.

[ref10] Farkas A. Németh R. (2022). How to measure gender bias in machine translation: real-world oriented machine translators, multiple reference points. Soc. Sci. Human. Open 5:100239.

[ref11] Foltynek T. Bjelobaba S. Glendinning I. Khan Z. R. Santos R. Pavletic P. . (2023). ENAI recommendations on the ethical use of artificial intelligence in education. Int. J. Educ. Integr. 19, 1–4.

[ref12] Gao R. Lin Y. Zhao N. Cai Z. G. (2024). Machine translation of Chinese classical poetry: a comparison among ChatGPT, Google translate, and DeepL translator. Human. Soc. Sci. Commun. 11, 1–10.

[ref13] Gaspari F. Almaghout H. Doherty S. (2015). A survey of machine translation competences: insights for translation technology educators and practitioners. Perspectives 23, 333–358.

[ref14] Goddard K. Roudsari A. Wyatt J. C. (2012). Automation bias: a systematic review of frequency, effect mediators, and mitigators. J. Am. Med. Inform. Assoc. 19, 121–127. doi: 10.1136/amiajnl-2011-000089, 21685142 PMC3240751

[ref15] Ibarra-Sáiz M. S. Gómez-Ruiz M. Á. Balderas A. Rodríguez-Gómez G. (2025). Improving learning through evaluative judgement and feedback using a technology-enhanced assessment environment. Technol. Knowledge Learn., 1–31.

[ref16] Jain R. (2024). “Assessing gender bias in machine translation” in In 2024 3rd international conference on applied artificial intelligence and computing (ICAAIC) (IEEE), 1091–1096.

[ref17] Karastergiou A. P. Diamantopoulos K. (2024). Gender issues in machine translation. Transcult. J. Human. Soc. Sci. 5, 48–64.

[ref18] King M. R. (2025). An update on ai hallucinations: not as bad as you remember or as you’ve been told. Cell. Mol. Bioeng., 1–6. 41328307 10.1007/s12195-025-00874-x PMC12664859

[ref19] Lee J. D. See K. A. (2004). Trust in automation: designing for appropriate reliance. Hum. Factors 46, 50–80, 15151155 10.1518/hfes.46.1.50_30392

[ref20] Maier-Hein L. Eisenmann M. Sarikaya D. März K. Collins T. Malpani A. . (2022). Surgical data science–from concepts toward clinical translation. Med. Image Anal. 76:102306. 34879287 10.1016/j.media.2021.102306 PMC9135051

[ref21] Mohamed Y. A. Khanan A. Bashir M. Mohamed A. H. H. Adiel M. A. Elsadig M. A. (2024). The impact of artificial intelligence on language translation: a review. Ieee Access 12, 25553–25579.

[ref22] Nowotny H. (2024). AI and the illusion of control. proceedings of the Paris Institute for Advanced Study, 1.

[ref23] Parasuraman R. Manzey D. H. (2010). Complacency and bias in human use of automation: an attentional integration. Hum. Factors 52, 381–410, 21077562 10.1177/0018720810376055

[ref24] Pedro F. (2012). Trusting the unknown: the effects of technology use in education. Global Inform. Technol. Rep.:2012.

[ref25] Prates M. O. Avelar P. H. Lamb L. C. (2020). Assessing gender bias in machine translation: a case study with google translate. Neural Comput. Applic. 32, 6363–6381.

[ref26] Ren X. (2025). We want but we can’t: measuring EFL translation majors’ intention to use ChatGPT in their translation practice. Human. Soc. Sci. Commun. 12, 1–11.

[ref27] Sahari Y. Al-Kadi A. M. T. Ali J. K. M. (2023). A cross sectional study of ChatGPT in translation: magnitude of use, attitudes, and uncertainties. J. Psycholinguist. Res. 52, 2937–2954. doi: 10.1007/s10936-023-10031-y, 37934302

[ref28] Sallam M. Elsayed W. Al-Shorbagy M. Barakat M. El Khatib S. Ghach W. . (2024). ChatGPT usage and attitudes are driven by perceptions of usefulness, ease of use, risks, and psycho-social impact: a study among university students in the UAE. Front. Educ. 9:1414758. doi: 10.3389/feduc.2024.1414758

[ref30] Sebastião S. P. Dias D. F. M. (2025). AI transparency: a conceptual, normative, and practical frame analysis. Media Commun. 13.

[ref31] Su J. Yang W. (2023). Unlocking the power of ChatGPT: a framework for applying generative AI in education. ECNU Rev. Educ. 6, 355–366.

[ref32] Wu Y. (2024). Critical thinking pedagogics Design in an era of ChatGPT and other AI tools—shifting from teaching “what” to teaching “why” and “how”. J. Educ. Develop. 8:1.

[ref33] Zhang Y. Liao Q. V. Bellamy R. K. (2020). Effect of confidence and explanation on accuracy and trust calibration in AI-assisted decision making. In Proceedings of the 2020 conference on fairness, accountability, and transparency (pp. 295–305).

